# Potentiality of multiple modalities for single-cell analyses to evaluate the tumor microenvironment in clinical specimens

**DOI:** 10.1038/s41598-020-79385-w

**Published:** 2021-01-11

**Authors:** Yukie Kashima, Yosuke Togashi, Shota Fukuoka, Takahiro Kamada, Takuma Irie, Ayako Suzuki, Yoshiaki Nakamura, Kohei Shitara, Tatsunori Minamide, Taku Yoshida, Naofumi Taoka, Tatsuya Kawase, Teiji Wada, Koichiro Inaki, Masataka Chihara, Yukihiko Ebisuno, Sakiyo Tsukamoto, Ryo Fujii, Akihiro Ohashi, Yutaka Suzuki, Katsuya Tsuchihara, Hiroyoshi Nishikawa, Toshihiko Doi

**Affiliations:** 1 Division of Translational Genomics, Exploratory Oncology Research & Clinical Trial Center, National Cancer Center, Chiba, 277-8577 Japan; 2 Division of Cancer Immunology, Exploratory Oncology Research & Clinical Trial Center, National Cancer Center, Chiba, Japan; 3 Department of Computational Biology and Medical Sciences, Graduate School of Frontier Sciences, The University of Tokyo, Chiba, Japan; 4 Department of Gastroenterology and Gastrointestinal Oncology, National Cancer Center Hospital East, Chiba, 277-8577 Japan; 5 Department of Gastroenterology and Endoscopy, National Cancer Center Hospital East, Chiba, Japan; 6 Drug Discovery Research, Astellas Pharma, Inc., Ibaraki, Japan; 7 Oncology Research Laboratories I, DaiichiSankyo. Co., Ltd., Tokyo, Japan; 8 Biomarker & Translational Research Department, DaiichiSankyo. Co., Ltd., Tokyo, Japan; 9 Takeda Pharmaceutical Company Ltd., Fujisawa, Japan; 10 Axcelead Drug Discovery Partners Inc., Fujisawa, Japan; 11 Division of Translational Informatics, Exploratory Oncology Research & Clinical Trial Center, National Cancer Center, Chiba, Japan; 12 Experimental Therapeutics, National Cancer Center Hospital East, Chiba, Japan

**Keywords:** Biological techniques, Cancer, Gastroenterology, Molecular medicine, Oncology

## Abstract

Single-cell level analysis is powerful tool to assess the heterogeneity of cellular components in tumor microenvironments (TME). In this study, we investigated immune-profiles using the single-cell analyses of endoscopically- or surgically-resected tumors, and peripheral blood mononuclear cells from gastric cancer patients. Furthermore, we technically characterized two distinct platforms of the single-cell analysis; RNA-seq-based analysis (scRNA-seq), and mass cytometry-based analysis (CyTOF), both of which are broadly embraced technologies. Our study revealed that the scRNA-seq analysis could cover a broader range of immune cells of TME in the biopsy-resected small samples of tumors, detecting even small subgroups of B cells or Treg cells in the tumors, although CyTOF could distinguish the specific populations in more depth. These findings demonstrate that scRNA-seq analysis is a highly-feasible platform for elucidating the complexity of TME in small biopsy tumors, which would provide a novel strategies to overcome a therapeutic difficulties against cancer heterogeneity in TME.

## Introduction

The complexity of tumors arises from various cellular components comprising the tumor microenvironment (TME); these include cancer cells, immune cells, fibroblasts, blood vessels, and the extracellular matrix^1^. Each cellular component also internally exhibits heterogeneous profiles with distinct morphology and phenotype, and the intra-tumor heterogeneity makes tumor contexture more complicated. Tumor heterogeneity, the diversity of cancer cell types in the tumor microenvironment, has recently attracted attention as a burgeoning research area, which is multi-directionally approached on the basis of cellular morphology, gene expression, metabolism, motility, proliferation, and metastatic potential. The interplay between the heterogeneous cancer cells with their microenvironments appears to play an important role in not only tumor development, but also therapeutic response/resistance to anticancer drugs^2,3^. Tumor-infiltrating lymphocytes (TILs) in TME, for instance, have a prominent role in determining the antitumor activity of immune checkpoint blockades (ICBs) such as anti-PD1 and anti-PD-L1 antibodies^4–9^. As for cancer cells, on the contrary, presentation of neo-antigens and/or PD-L1 expression on the surface of cancer cells apparently contribute to the efficacy of ICBs^10,11^. Given that cancer cells exhibit heterogeneous expression levels of these factors, the use of these factors as biomarkers requires further improvements for efficacious predictive precision^12–14^. To capture a view of tumor complexity more comprehensively and panoramically, single cell analysis, rather than a conventional bulk analysis, is expected to be a powerful tool since this methodology could profile small heterogeneous populations in the tumor microenvironment (TME)^15,16^.

For decades, flow cytometry is the most widely-used methodology to analyze single cells, especially immune cells. A novel research platform of flow cytometry equipped with mass spectrometry, termed mass cytometry (CyTOF), has been recently developed. CyTOF could detect single-cell resolution using ~ 40 simultaneous cellular parameters, which evaluates the complexity of cellular systems and processes^17–19^. Although single-cell analysis with CyTOF has been well-established, especially for the analysis of peripheral blood mononuclear cells (PBMCs)^20,21^, it also possesses technical difficulties to overcome. Firstly, analysis for TIL in a small biopsy sample is technically challenging. Secondly, the number of measured parameters is limited (~ 40 parameters), indicating that only focused cell populations can be detected by CyTOF^18^. On the contrary, single-cell RNA-sequencing (scRNA-seq) is based on the expression level of the entire gene in individual cells and is expected to cover various biological pathways comprehensively^22,23^. Recently, a droplet-based scRNA-seq successfully characterized various clusters of immune cells based on the gene expression profile; this methodology is gaining popularity for widespread use^24,25^.

In this study, we investigated immune-profiles using the single-cell analyses of clinical specimens, endoscopically- or surgically-resected tumors, as well as PBMCs from gastroenterological cancer patients. Furthermore, we technically characterized two distinct platforms of the single-cell analysis, 10 × Genomics Chromium Single cell 3′ v2-based scRNA-seq analysis and Fluidigm Helios-based CyTOF analysis, both of which are broadly embraced technologies for single-cell level analysis. Using the two-distinct methodologies, we demonstrated that single-cell analysis is a powerful tool for classifying the cell types in clinical specimens as well as to understand the complexity of the TME.

## Results

### Evaluation of TILs in TME by CyTOF analysis, comparing fresh and frozen tumors

**Figure 1:**
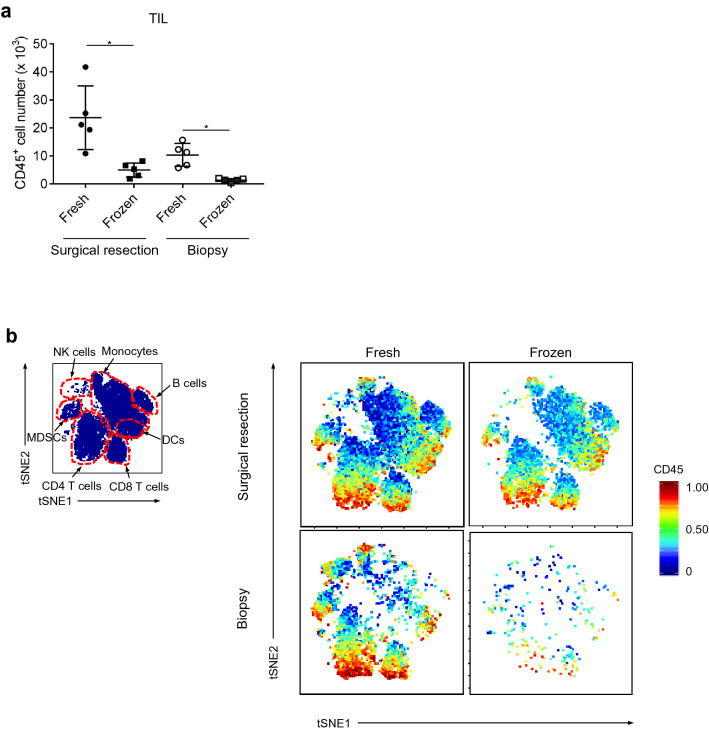
Freshly isolated cells showed better viability and detection compared with frozen samples in CyTOF. (**a**) The number of CD45 cells identified from the fresh and frozen surgically- and biopsy-resected samples. (**b**) Their representative staining figures. Fractions with approximately 5 mm square size from surgically resected samples or three fractions from biopsy samples were used for CyTOF. After sampling, the tumor tissues were minced by gentleMACS. Half of the cell suspension was applied for CyTOF at the fresh state, and the remaining one was stocked, which was analyzed a few weeks later. *p < 0.05.

We first conducted single-cell analysis in Fluidigm Helios-based CyTOF to evaluate and compare TILs isolated from freshly-prepared and frozen-stocked tumor samples. For this analysis, surgically-resected gastrointestinal cancer specimens were used (Sup. Table S1 and S2: patient). As shown in Fig. 1a, we detected significantly higher cell numbers, as well as higher numbers of marker-positive cells, from the fresh samples than the frozen samples (Fig. 1b; upper panel). Similar to the surgically-resected samples, the endoscopically-resected biopsy samples (Sup Table S1) also exhibited higher cell numbers (Fig. 1a) and more marker-positive cells (Fig. 1b; lower panel) in the fresh samples than the frozen samples, revealing that CyTOF analysis of freshly-prepared samples worked better for both surgically-resected and endoscopically-resected specimens. These results indicate that freshly-prepared tumors would be preferred for single-cell analyses to evaluate TILs in TME, especially for analyses of small pieces of the biopsy samples.

### Evaluation of two distinct platforms for single-cell analysis in PBMCs: scRNA-seq and CyTOF

**Figure 2:**
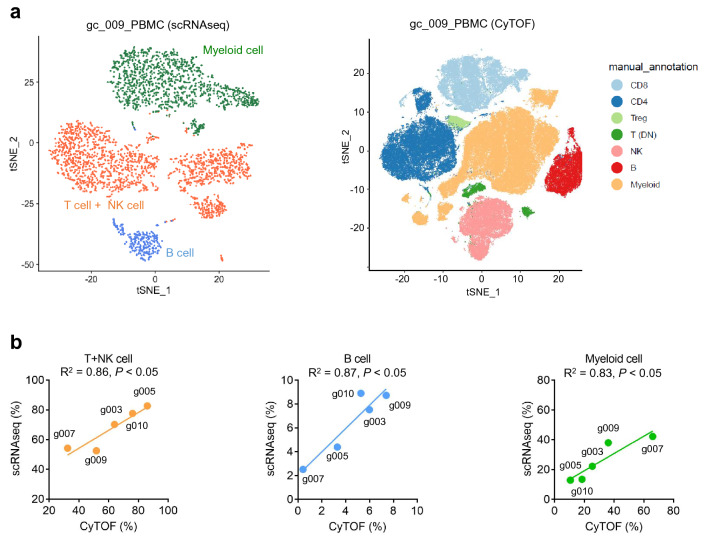
Comparison between scRNA-seq data using a droplet-based system and CyTOF data showed good concordances in PBMCs. (**a**) t-SNE plot based on scRNA-seq (left) and t-SNE plot based on CyTOF (right) of PBMCs. The same samples were divided into two groups for each method. After clustering, cells are annotated using representative genes or molecules in Table S3 (for CyTOF) and Table S5 (for scRNA-seq). (**b**). Comparison between CyTOF data (x-axis) and scRNA-seq data (y-axis). %T + NK cell, %B cell and %Myeloid cell in CD45 + immune cells are presented.

We evaluated two distinct platforms for single-cell analysis: Chromium v2 (10X Genomics) for scRNA-seq and Fluidigm Helios-based CyTOF using the same PBMC sample sets. R package Seurat for scRNA-seq and CytoBank for CyTOF were used to classify PBMC populations based on the indicated markers (Figs. 2a, S1, S2 and Sup Table S3 and S4) ^26,27^. Both scRNA-seq and CyTOF clearly distinguished the classified immune-cell types such as T cells, NK cells, B cells, and other immune-related cells in PBMCs. However, scRNA-seq exhibited less accuracy to identify the differences between T cells and NK cells (Fig. 2a, S1, S2 and Sup Table S4). We also compared the abilities of scRNA-seq and CyTOF single-cell analysis to detect % T + NK cell, % B cell, and % myeloid cell in CD45^+^ immune cell populations, revealing similar proficiencies between these platforms. The values of coefficient of determination (R^2^) were 0.86 in T + NK cells, 0.87 in B cells, and 0.83 in myeloid cells (Fig. 2b). However, one sample (gc_007) showed poor agreement between the techniques, which could be due to a technical error in the recovery from the frozen stock, since cell viability of this sample was extremely low (Fig. S3a).

### Comparison of single-cell analyses between scRNA-seq and CyTOF in the endoscopically-resected biopsy samples

**Figure 3:**
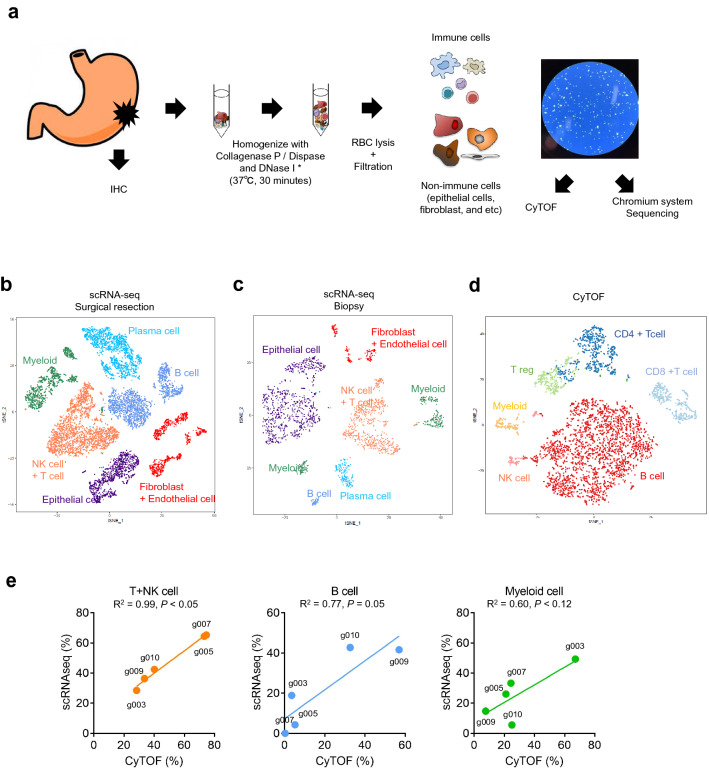
ScRNA-seq using whole tissues could reveal TME. (**a**) The procedure of sample processing. We marked the location of sampling for CyTOF and scRNA-seq. When IHC was performed, we used the marked blocks. Fractions with approximately 5 mm square size from surgically resected samples were used for CyTOF and scRNA-seq. After sampling, tumor tissues were minced, and then isolated using the dissociator. Enzyme treatments were used for scRNA-seq for 30 min at 37 °C (*). The isolated cell suspension was subjected to CyTOF and scRNA-seq at the fresh state. (**b**–**d**) t-SNE plots based on scRNA-seq of surgical resection (**b**), biopsy sample (**c**), and on CyTOF of surgical resection (**d**). (**e**) Comparison between CyTOF (x-axis) data and scRNA-seq data (y-axis) in each immune-cell population. %T + NK cell, %B cell, and %Myeloid cell in CD45 + immune cells are presented.

Compared to the surgically-resected tumor samples, the endoscopically-resected tumor biopsy samples were much smaller. Thus, to utilize these small numbers of cells in the biopsies as much as possible, we subjected the whole biopsy tumors with no sorting to the single-cell analyses for scRNA-seq and CyTOF. In this manner, we expected to mitigate the loss of cell numbers that occurs when using small pieces of the biopsies. The unsorted whole tumors in the biopsies should include not only immune cells, but also non-immune cells, such as epithelial cells, fibroblasts, endothelial cells, and other TME-component cells. As a preliminary study, we first performed scRNA-seq analyses using this unsorted methodology on the surgically-resected tumors (Fig. 3a). Freshly-prepared tumors were used for the analyses, and the experiments were quickly initiated within 30 min after surgical resection. The single cells in the tumors were isolated by treatment with the indicated dissociation enzyme and then directly, without the sorting step, subjected to Chromium v2 scRNA-seq analyses. The gene expression analyses of the scRNA-seq by Seurat clearly identified a number of clusters of the TME components in the surgical tumors—NK + T cells, B cells, plasma cells, myeloid cells, epithelial cells, and fibroblast + endothelial cells (Fig. 3b)—revealing that this scRNA-seq with “no sorting” protocol could clearly detect the clusters of TIL components even though the unsorted tumors would contain various non-immune cells (Figs. 3b, Fig. S4, and Table S4).

Next, we conducted scRNA-seq analyses of the endoscopically-resected tumor biopsies. For this study, we also used freshly-prepared biopsies with the “unsorted protocol” as established above. As shown in Fig. 3c, the gene expression analyses by Seurat clearly identified a number of clusters of the TME components in the small biopsy samples as well, namely NK + T cells, B cells, plasma cells, myeloid cells, epithelial cells, and fibroblast + endothelial cells. Taken together, the single-cell isolation protocol in unsorted tumors technically works to evaluate the clusters of TIL components in the endoscopically-resected tumor biopsies.

We also evaluated concordance between the scRNA-seq and CyTOF results in distinguishing % T + NK cells, % B cells, and % myeloid cells in the surgically-resected tumors (Fig. 3e). The values of coefficient of determination (R^2^) were 0.99 in T + NK cells, 0.77 in B cells, and 0.60 in myeloid cells. Although the R^2^ was relatively low in B and myeloid cells, the correlative results between the two platforms were broadly confirmed in tumors as well as PBMCs (Figs. 2 and 3). Interestingly, scRNA-seq identified the plasma cells, a CD45^-^CD138^+^IGKC^+^CD20^-^ cluster, while CyTOF did not due to lack of B cell markers in our preset CyTOF panel (only CD20 was represented). Given that the markers of CyTOF must be selected for the target(s) prior to the experiments, this may be a technical limitation of CyTOF to cover a broader range of (unbiased) heterogeneous populations.

### Comparison between scRNA-seq data and IHC or CyTOF data

**Figure 4:**
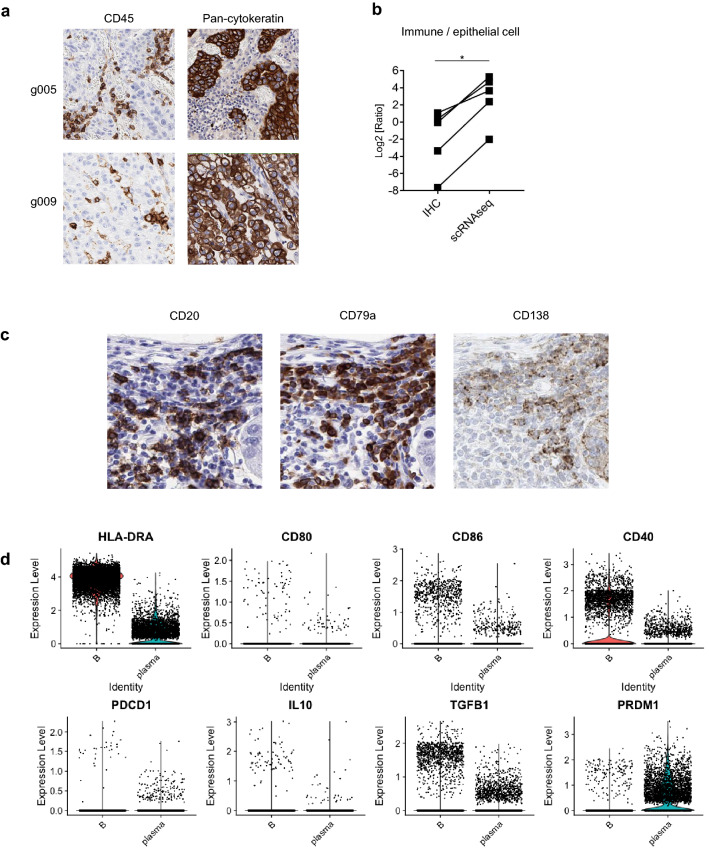
Comparison between scRNA-seq data using a droplet-based system and CyTOF data showed a little worse concordances in the TME. (**a**) Representative Immunohistochemistry (IHC) analysis for CD45 (left) and pan-cytokeratin (right). (**b**) Comparison between IHC and scRNA-seq with the ratio of epithelial cells / immune cells. Sample processing was performed as described in Fig. 3a. *p < 0.05. (**c**) Representative IHC analysis for plasma cells (CD138 + CD79a + CD20− cells). (**d**) Violin plots for comparison between tumor-infiltrating B cells and plasma cells based on scRNA-seq data. Antigen-presenting related genes (top) and regulatory B-cell related genes (bottom) are presented.

Next, we evaluated the concordance between scRNA-seq and IHC data. The location of sampling area for CyTOF and scRNA-seq were marked, and the marked blocks were stained for IHC (Fig. 4a). We analyzed the ratio of immune cells/epithelial cells by IHC staining of CD45 and pan-cytokeratin and compared them with the scRNA-seq data (Fig. 4b). While a similar tendency was observed, the immune cell ratio was remarkably high in scRNA-seq data compared with that in IHC data, indicating that a considerable number of epithelial cells, which include cancer cells, can be damaged and lost during the procedure. Next, to evaluate further detailed concordance, we compared scRNA-seq data with CyTOF data, focusing on immune cells and observed poor concordances in some samples (Fig. S3b). Populations of B cell and myeloid cell had poor concordances as well (Fig. 3e). These findings suggest that there are the technical limitations of the experimental procedure in addition to data analyses, and its influence on the analysis of tumor tissue is greater as compared with PBMC (Fig. S3).

As with the result by scRNA-seq data (Fig. 3b), CD138^+^CD79a^+^CD20^-^ plasma cells were also identified by IHC (Fig. 4c). Tumor-infiltrating B cells highly expressed HLA class II and CD40 (Fig. 4d), were also identified, suggesting these cells play as antigen-presenting cells. A fraction of B cells expressed PDCD1, IL10, and TGFB1. In addition, PRDM1 was also expressed, similarly to plasma cells (Fig. 4d). These suggest that regulatory B cells infiltrated into the TME^28^. We also found this plasma-cell population in biopsy samples by scRNA-seq (Fig. 3c). Taken together, our established scRNA-seq technique enabled us to find novel cell populations, although there were some technical limitations.

### Classification of regulatory T cells (Treg cells) in TILs

**Figure 5:**
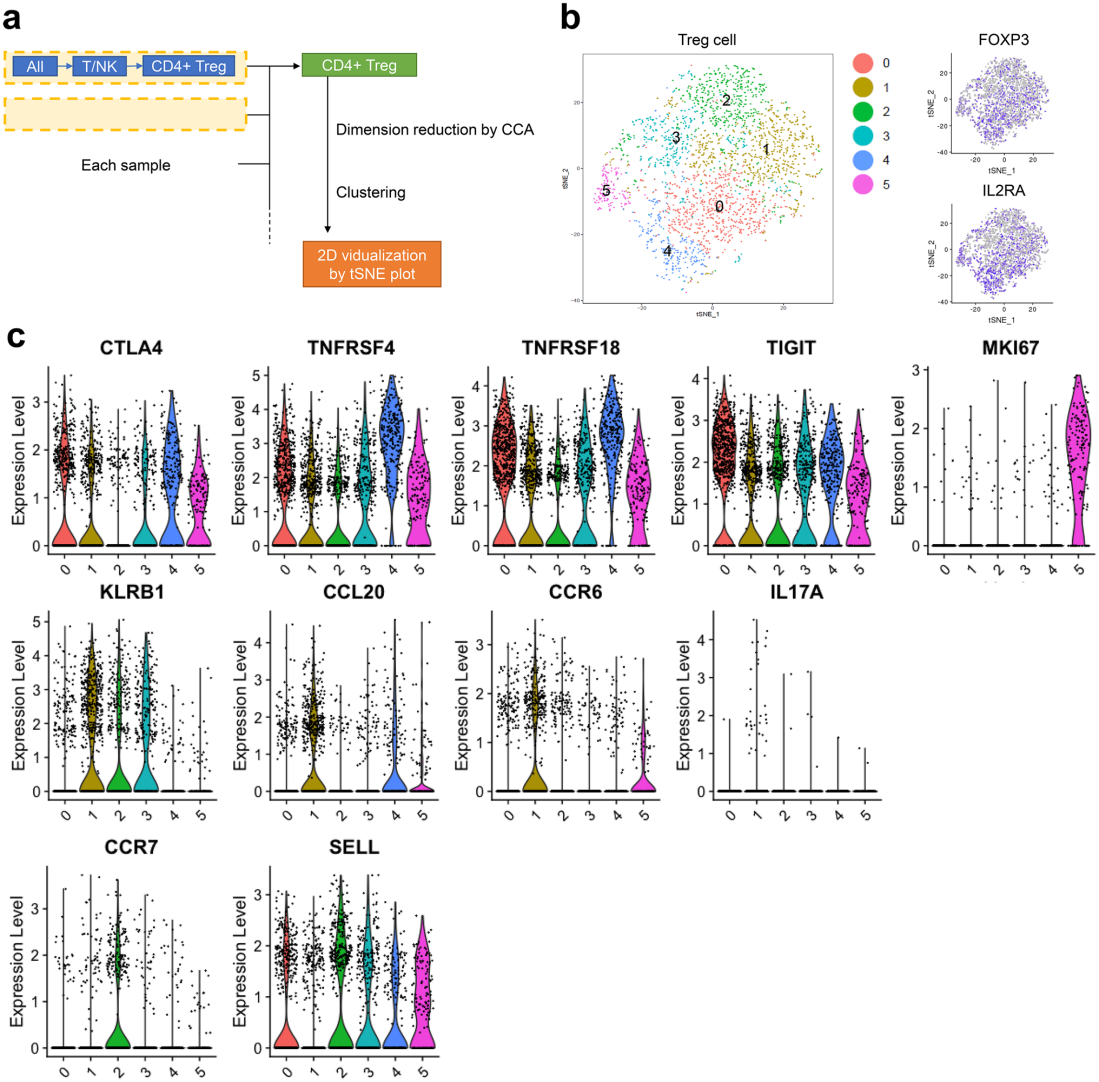
ScRNA-seq revealed Treg cells in the TME have heterogeneity. (**a**) The procedures followed for the data processing. Briefly, the Treg-cell population was extracted from each sample as FOXP3 + CD25 + CD4 + T cells, concatenated, and analyzed. (**b**) t-SNE plot (left) and gene expression feature plot for Foxp3 (right, top) and IL2RA (right, bottom) of concatenated Treg cells. Six clusters of Treg cells were identified in the TME. (**c**) Violin plots showing the gene expression of Treg cells in the TME of representative genes.

To investigate the detail of Treg function, T + NK-cell scRNA-seq datasets were re-analyzed (Fig. 5a). Treg datasets were extracted based on FOXP3 and IL2RA gene expressions among CD4^+^ T cells (Fig. 5b). As in Fig. 5b, we classified the Treg cell into 6 clusters. Top 10 genes (average log2 fold change > 0.5 compare to other clusters and adjusted p-value < 0.05) are summarized in Sup Table S6. Cluster 0 and 4 (35.5% ± 4.7%) were characterized by CTLA4, TNFRSF4, TNFRSF18, and TIGIT, which are highly expressed by activated Treg cells in general (Fig. 5c)^29^. By contrast, these molecules were low, and MKI67 were highly expressed by cluster 5 cells (7.3% ± 4.9%), which were considered as proliferative Treg cells (Fig. 5c). Treg cells in cluster 1 (24.1% ± 4.9%) expressed KLRB1 (CD161) and CCL20, which are specific for Th17 (Fig. 5c). Accordingly, CCR6, a receptor of CCL20, and IL17A were also highly expressed by this cluster (Fig. 5c). Thus, this cluster indicated CD161^+^ Th17-like Treg cells produce proinflammatory cytokines. CCR7 and SELL (CD62L) were highly expressed by cluster 2 cells (19.0% ± 7.1%), which were considered as naïve Treg cells (Fig. 5c). Cluster 3-specific molecules, including IFI44L, STAT1, ISG15, and IFITM1, are generally induced by interferon response, suggesting that cluster 3 cells (14.1% ± 5.1%) are interferon-related Treg cell (Sup Table. S6). STAT4 did not express by all clusters, including cluster 3, as previously reported (Fig. S6)^30^.

## Discussion

In this study, we evaluated the two distinct single-cell-based platforms, scRNA-seq and CyTOF, for single-cell level immune profiling using surgery-/biopsy-resected tumors and PBMC from gastric cancer patients. The number of previous studies focusing on gastric cancer biopsy in single-cell level is limited^31–33^. Our study revealed that the scRNA-seq analysis could cover a broader range of immune cells of TME in the endoscopically-resected small biopsy tumor samples, detecting even small subgroups of B cells or Treg cells in the tumors. These findings demonstrate that scRNA-seq analysis is a highly-feasible platform for elucidating the complexity of the tumor microenvironments in small biopsy tumors.

Recent advances in single-cell level analysis, such as scRNA-seq and CyTOF, allowed us to investigate the complexity of heterogeneity in TME in more detail, detecting small heterogeneous populations (e.g., TILs) at a high dimensional level^34,35^; however, each of these platforms has strengths and weaknesses in their technical performance^36,37^. CyTOF, which has an advantage in higher throughput compared to scRNA-seq, could detect the targeting immune cell subsets more clearly using the ~ 40 selected antigen markers^19,38^. However, since these markers must be selected for target detection prior to experiments, the parameters that CyTOF can measure are technically limited to cover a broader range of heterogeneous populations^18^. In addition, there is the technical challenge of generating marker antibodies conjugated with metal-isotopes, which also results in narrowing down the populations that CyTOF can detect. On the contrary, scRNA-seq is a transcriptome-based platform, which detects wider unbiased-populations without marker selection. However, one limitation of droplet-based scRNA-seq is that its transcription-based data are relatively shallow and sparse^39,40^; the numbers of transcriptomes in every single cell are in the range of several thousands. Our study also demonstrated the features of these two distinct platforms clearly: CyTOF is “narrow and clear,” whereas scRNA-seq is “wide and indistinct.” CyTOF distinguished NK cells and T cells more accurately, while scRNA-seq detected plasma cells that CyTOF markers did not cover (Figs. 2 and 3). Taken together, we need to choose the best platform of single-cell analysis for the purpose and/or the combination of multiple platforms to compensate for their individual limitations.

We also optimized and established the protocols of scRNA-seq analysis for small clinical specimens of the biopsy-resected tumors. The same procedure used for the surgically-resected tumors could be applied to the protocols for the endoscopically-resected small tumor biopsies as well. Keys to success for single-cell analysis in the small biopsies appear to be (1) to use freshly-prepared tumor biopsies, and (2) to run the unsorted cells for the analysis. We initiated the single cell isolation as quickly as possible (~ 30 min) after receiving the tumor samples. The “no sorting” technique appears to mitigate physical damage to the primary cells, thus making it more suitable for analyses of small numbers of cells.

The optimized protocols allowed us to identify small heterogeneous populations of Treg in TME, suggesting the scRNA-seq analysis appears to be a highly-feasible platform to understand the complexity of the tumor microenvironments in the small biopsy tumors (Fig. 5). A number of scRNA-seq studies in the surgery-resected clinical specimens have been reported, providing a large amount of comprehensive information to deeply understand the clinically-relevant cancer biology, invasion, metastasis, and cancer evolution^41^. However, since the surgery-resected tumors are basically at the earlier stages of tumor development, the information obtained from the surgery-resected tumors may be restricted to the earlier-stage biological events of tumorigenesis, presumably missing the later-stage events. The small pieces of biopsy-resected tumors, on the contrary, could be technically collected from the later-stage cancer patients; thus, the single-cell analysis using these biopsy-resected tumors is expected to cover the valuable information from later-stage cancer. In addition, if the biopsy samples could be collected before and after the drug treatment in the same patients, the single-cell analysis with these pre-/post-treatment specimens should provide a great advantage for a deep understanding of the mechanisms of actions and/or biomarker development for cancer therapeutic drugs^41–43^. In fact, the single-cell analyses in the biopsy samples, including the scRNA-seq analysis in gastric cancer, have received a lot of attention in recent years, while the number of reports is extremely limited^31–33^. Although several technical challenges still need to be cleared for scRNA-seq of the biopsy-resected tumors, especially the cell isolation steps to miss certain cell populations by “bottle-neck effects”^33,44^, this research platform of scRNA-seq should have potential to open the doors to the new generation of cancer biology, overcoming a number of difficulties that currently-used conventional methodologies are facing.

In conclusion, we demonstrated two-distinct methodologies for single-cell analysis as powerful tools to clarify the subpopulations of clinical specimens. Although deeper analysis is required, the methods of the single-cell analysis showed the potential to identify various cell populations, which could not be identified by other modalities providing novel insights into the tumor microenvironment. Taken with the technical advantages for each methodology, the single-cell analysis would be more powerful tools to understand the complexity of the TME.

## Methods

### Patients

Patients with gastrointestinal cancer, who underwent surgical resection or endoscopic biopsy at National Cancer Center Hospital East in 2017, were enrolled in this study (Sup Table S1 and S2). All patients provided written informed consent before sampling, according to the Declaration of Helsinki. This study was performed in a blinded manner and was approved by the National Cancer Center Ethics Committee.

### The procedure of sample processing

#### Tumor sample processing

Single fraction (~ 5 mm square size) from surgically-resected tumor samples or three fractions (~ 3 mm square size for each) from endoscopically-resected tumor biopsy samples were subjected to CyTOF and scRNA-seq analyses. The freshly resected surgery or biopsy samples were kept in ~ 3 ml of cold saline solution, to start single-cell isolation in ~ 30 min after the tumor dissection. The tumor samples were substantially minced with a surgical blade and scissor into small pieces, and then isolated into single cells using gentleMACS Dissociator (Miltenyi Biotec, Bergisch Gladbach, Germany) at 37 °C for 30 min digesting with ~ 5 ml of enzyme mixture, which includes 4 ml of DMEM medium with 10% FBS (DMEM/FBS), 1 ml of Collagenase P (final conc. 2 mg/ml, cat# 11213865001, Merck KGaA, Darmstadt, Germany) or Dispase (final conc. 2.5 mg/ml, cat# 4942078001, Merck KGaA, Darmstadt, Germany), and 50 μl of DNase I (final conc. 0.1 mg/ml, Qiagen, Venlo, Netherlands). The digested tumors were filtrated through 40 μm and 100 μm nylon mesh to remove cell aggregates, and cell viability was determined by microscopy (> 80% viability is preferable). Collecting the cell pellets by spin-down at 300 g for 10 min at 4 °C, the cells were suspended with 1 ml of Red Blood Cell Lysis Solution (cat# 130-094-183, Miltenyi Biotec B.V. & Co. KG, Bergisch Gladbach, Germany). After incubation for 2 min at 4 °C, the cells were suspended with 20 ml of DMEM/FBS, spinning down the cell pellets at 300 g for 5 min at 4 °C. The cells were substantially suspended in 1 ml of PBS with 10% FBS (PBS/FBS) and were filtrated through 40-μm nylon mesh. The resuspended cells with PBS/FBS at 1 × 10^6^ cell/ml were subjected to the single-cell analysis. The remaining portion of the isolated tumors were stocked in CELLBANKER (cat# CB011, Nippon Zenyaku Kogyo, Tokyo, Japan) according to the manufacturer’s instruction, which were used as “frozen samples”.

#### PBMC sample processing

PBMC isolation by density gradient centrifugation with Ficoll-Paque was performed according to the manufacturer’s instruction (cat#17-1440-03, GE Healthcare Bio-Science AB, Uppsala, Sweden). Briefly, 4 ml of blood samples were carefully layered on to 3 ml of Ficoll-Paque media to the centrifuge tube, and then centrifuged at 400×*g* for 30 min at room temperature. The PBMCs were collected from the interface layer. After washing with DMEM/FBS, PBMCs were suspended in 1 ml of PBS/FBS and were filtrated through 40-um nylon mesh. The resuspended cells with PBS/FBS at 1 × 10^6^ cell/ml were subjected to the single-cell analysis.

### IHC

Surgically resected samples were formalin-fixed, paraffin-embedded, and the blocks which we marked before sampling for CyTOF and scRNA-seq were sectioned onto slides for IHC, which was conducted using monoclonal antibodies against CD20 (L26, Roche, Basel, Switzerland), CD45 (2B11 + PD7/26, DAKO, Agilent Technologies, Santa Clara, CA the USA), pan-cytokeratin (AE1, AE3, PCK26, Roche, Basel, Switzerland), CD79a (SP18, Roche, Basel, Switzerland), and CD138 (M115, DAKO, Agilent Technologies, Santa Clara, CA USA). CD45 and pan-cytokeratin staining were counted in five high-power microscopic fields (× 400; 0.0625 mm^2^), and their averages were calculated. Two researchers (Y.T. and T.K.) independently evaluated the stained slides.

### CyTOF procedure

CyTOF staining and analysis were performed as described^20^. The antibodies used in CyTOF analyses are summarized in Table S3. The cells were subjected to staining after washing with PBS supplemented with 2% fetal calf serum (FCS, Biosera, Orange, CA, USA) (washing solution) followed by incubation in 5 μM of Cell-ID rhodium solution (Fluidigm, South San Francisco, CA, USA) in PBS, washed using the washing solution, and stained with a mixture of surface antibodies. After washing, the cells were fixed and permeabilized using Foxp3/Transcription Factor Staining Buffer Set (Thermo Fisher Scientific, Waltham, MA) according to the manufacturer’s instructions. The fixed and permeabilized cells were stained with intracellular antibodies. After washing twice, the cells were incubated overnight in 125 nM MaxPar Intercalator-Ir (Fluidigm) diluted in 2% paraformaldehyde PBS solution at 4 °C. The cells were then washed once with the washing solution and twice with MaxPar water (Fluidigm), distilled water with minimal heavy element contamination, to reduce the background level. The cells were then suspended in MaxPar water supplemented with 10% EQ. Four Element Calibration Beads (Fluidigm) were applied to the Helios instrument (Fluidigm), and data were acquired at speed below 300 events/s.

### CyTOF data processing

Using Cytobank^45^, a manual-gating scheme was processed to remove doublet cells, dead cells, and beads. After the cleanup processes, multidimensional data were clustered using R package FlowSOM^46^ and reduced dimension using R package Rtsne. After the visualization, cells were annotated by the expression of the following representative cell surface markers; T cell (CD3+ and CD8a+, or CD3+ and CD4+), B cell (CD19+), NK cell (CD56+), and myeloid cell (CD11b+ or CD11c+).

### scRNA-seq procedure

Samples were processed using the Chromium Single Cell 3′ Solution v2 chemistry (10 × Genomics, CA, USA) as per the manufacturer’s recommendations^24^. Briefly, cell suspension is resuspendeed at 1 × 10^6^ cells per ml. To generate GEMs, master mix with cell suspension, gel beads and partioning oils are loaded on Chromium Chip. GEM-RT reaction, cDNA amplification, gene expression library generation were followed using Chromium kits and reagents. After library generation, sequencing was performed using Illumina HiSeq 2500 Rapid run with 98-bp pair-end reads. Using Cell Ranger (version 2.0, 10 × Genomics), the fastq files were generated from the bcl files. The sequence reads were aligned to UCSC hg38 and UMIs (Unique Molecular Identifiers) were counted for each gene in each cell barcode using Cell Ranger count (option: –expect_cells = 6000). Then, the data were polished by R package Seurat as below^26,27^.

### scRNA-seq data processing for PBMC samples

Using Cell Ranger output files, barcodes.tsv, genes.tsv, and matrix.mtx, Seurat objects were created. Cells with UMI < 1500, expressing < 100 genes, and with > 10% mitochondrial genes were removed from PBMC datasets using Seurat v2.3.4. Then, the UMI counts were normalized and scaled. Clustering and 2D projection by t-distributed Stochastic Neighbor Embedding (t-SNE), was also performed after dimensional reduction using the first 10 Principal Components (PCs). For the feature plot, the dataset was updated and plotted using Seurat v3. The number of cells after the process was shown in Table S5. Cells were annotated with cell types by the expression levels of 40 markers in Table S4.

### scRNA-seq data processing for TIL samples

Using Cell Ranger output files, barcodes.tsv, genes.tsv, and matrix.mtx, Seurat objects were created. The number of cells with UMI ≥ 1500 were counted using the gene-cell matrix of Cell Ranger. To extract the data of single cells with UMI ≥ 1500, Cell Ranger count was re-conducted with the option “–force_cells” with the number of cells with UMIs ≥ 1500 in each sample. Then, the data was aggregated using Cell Ranger aggr with the option “normalize = none” for combining the data from the same patients. Using Seurat v2.3.4, cells with > 10% mitochondrial genes and expressing < 500 genes were discarded from the datasets. The total reads and the number of detected cells were shown in Table S7. Then, the UMI counts were normalized and scaled. Clustering and 2D projection by t-SNE were also performed after dimensional reduction using the first 20 PCs using Seurat. Using representative marker genes of each cell type in Table S4, the cell clusters were annotated^22^.

As gc_003 differs from other samples in total reads, it was removed for the analysis of Figs. 4d and 5.

For the analysis of Fig. 4d, the cell clusters, which were defined as B or plasma cells, were extracted from four patients and individually gathered into B-cell and plasma-cell groups. Then, the expression levels of the representative B-cell and plasma-cell related genes were plotted using Seurat v3.

For the analysis of Fig. 5, the cell clusters, which were defined as regulatory T cells, were extracted from the datasets of each patient. Briefly, T + NK clusters were first extracted from the TIL datasets and the T + NK cells were re-clustered after cell cycle regression and dimensional reduction using the first eight PCs. Then, clusters were extracted according to the expression of CD3, CD4, and FOXP3 as Tregs. The extracted Treg clusters of all the cases were combined using the first eight PCs by performing Seurat RunMultiCCA and AlignSubspace. The Tregs were re-clustered into six sub-clusters (clusters 0–5). For each cluster, the top 10 marker genes were identified, as shown in Table S6.

## Supplementary Information

Supplementary Legends.

Supplementary Figure 1.

Supplementary Figure 2.

Supplementary Figure 3.

Supplementary Figure 4.

Supplementary Figure 5.

Supplementary Figure 6.

Supplementary Table 1.

Supplementary Table 2.

Supplementary Table 3.

Supplementary Table 4.

Supplementary Table 5.

Supplementary Table 6.

Supplementary Table 7.

## Data Availability

The datasets generated and/or analyzed during the current study will be available on Database of National Biosceience Database Center before this manuscript is published.
