# Supplementary material for: Potentiality of multiple modalities for single-cell analyses to evaluate the tumor microenvironment in clinical specimens
Source: Sci Rep. 2021 Jan 11;11:341. doi: 10.1038/s41598-020-79385-w (PMC7801605; doi:10.1038/s41598-020-79385-w)
Supplement: Supplementary file 1 — Supplementary Legends. [file 41598_2020_79385_MOESM1_ESM.docx]

**Supplementary Figure S1.** Specific gene expression feature plot for annotation based on scRNA-seq data of PBMCs.

**Supplementary Figure S2.** Specific molecule expressions for annotation in t-SNE plot based on CyTOF data of PBMCs

**Supplementary Figure S3.** Concordances in each patient between scRNA-seq data and CyTOF data in PBMCs (a) and TILs (b). Orange, T+NK cell; blue, B cell; green, myeloid cell.

**Supplementary Figure S4.** Specific gene expression feature plot for annotation based on scRNA-seq data of TME.

**Supplementary Figure S5.** Specific molecule expressions for annotation in the t-SNE plot based on CyTOF data of TME.

**Supplementary Figure S6.** Violin plots showing STAT1 and STAT4 expressions of Treg cells in the TME

**Sup Table S1.** Information of clinical samples used in Fig.1

**Sup Table S2.** Information of clinical samples used in Fig.2, 3, 4, and 5

**Sup Table S3.** Antibodies used in the CyTOF

**Sup Table S4.** gene list used to annotate cell type

**Sup Table S5.** scRNA-seq (PBMC)

**Sup Table S6.** Differentially expressed genes in each Treg clusters in Figure 5b

**Sup Table S7.** Stats of TIL scRNA-seq
