# Supplementary figures and images for: Potentiality of multiple modalities for single-cell analyses to evaluate the tumor microenvironment in clinical specimens
Source: Sci Rep. 2021 Jan 11;11:341. doi: 10.1038/s41598-020-79385-w (PMC7801605; doi:10.1038/s41598-020-79385-w)

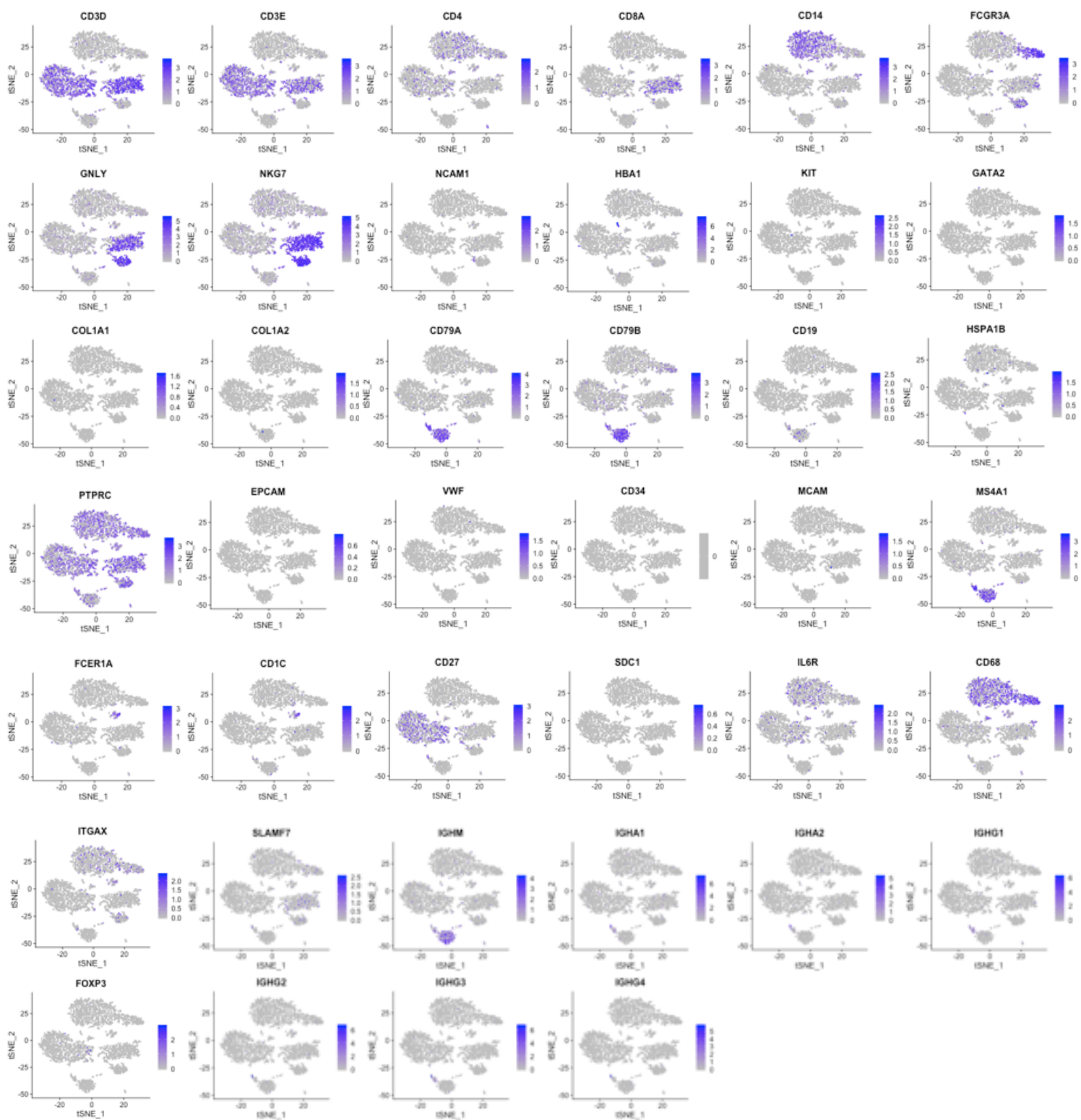

Figure S1

Supplement: Supplementary file 2 — Supplementary Figure 1. [file 41598_2020_79385_MOESM2_ESM.pdf]

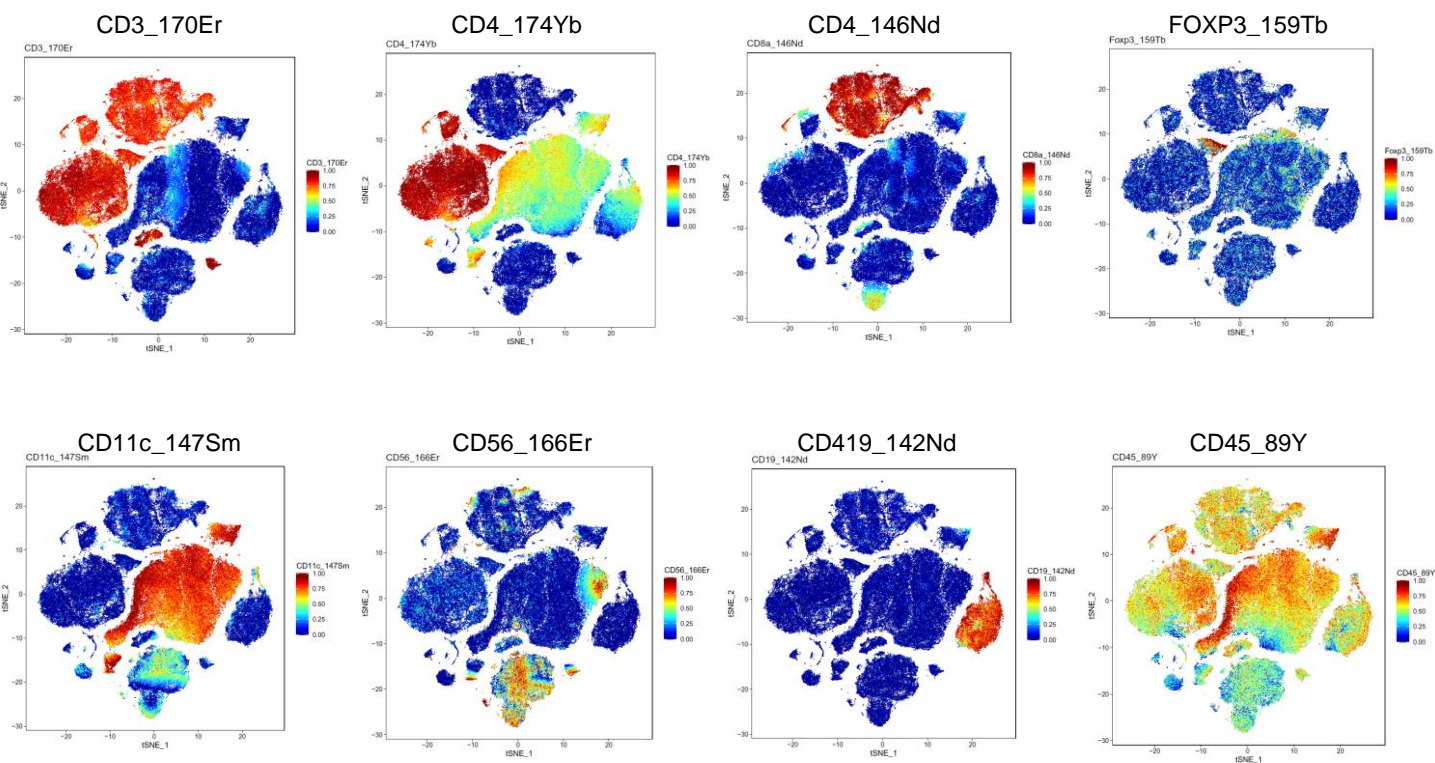

Figure S2

Supplement: Supplementary file 3 — Supplementary Figure 2. [file 41598_2020_79385_MOESM3_ESM.pdf]

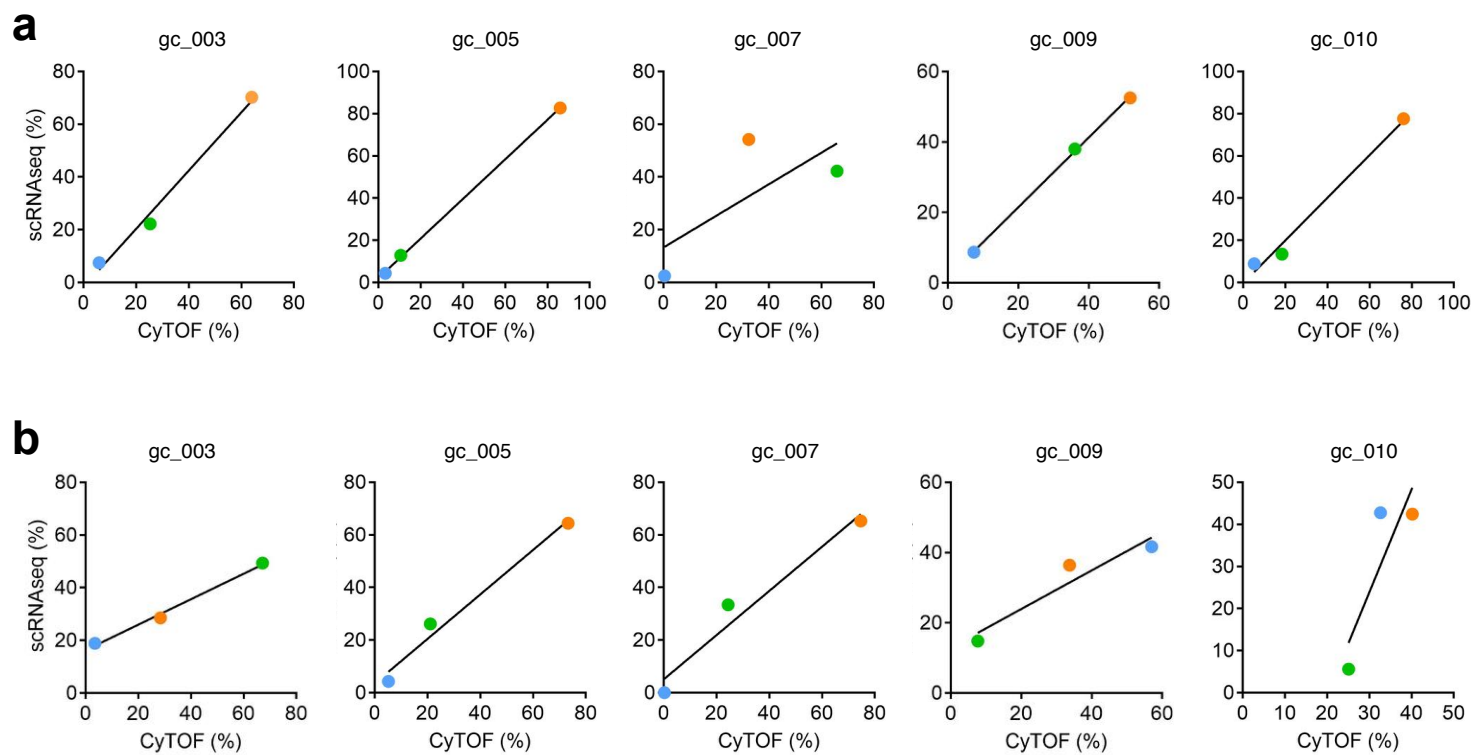

Figure S3

Supplement: Supplementary file 4 — Supplementary Figure 3. [file 41598_2020_79385_MOESM4_ESM.pdf]

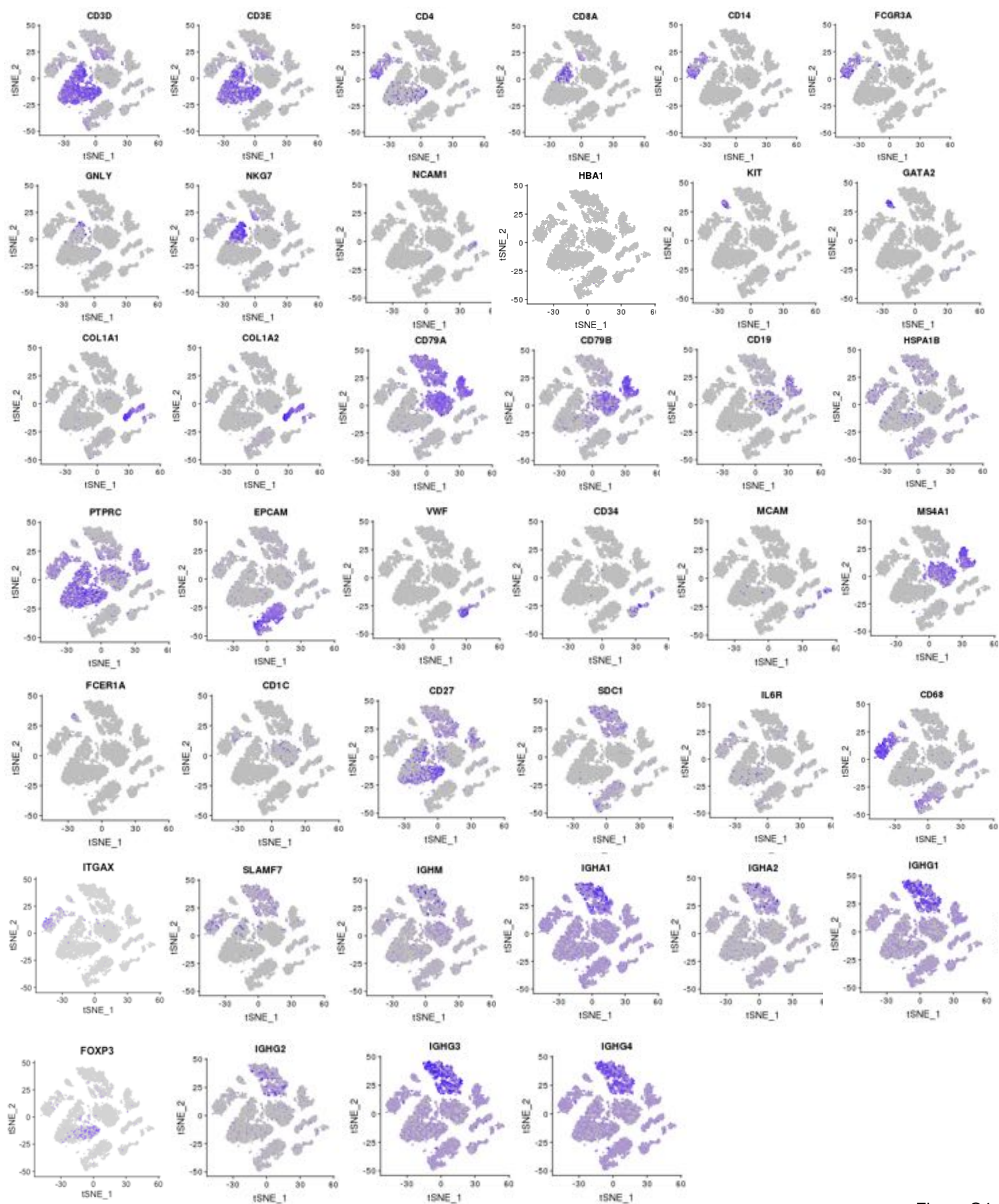

Figure S4

Supplement: Supplementary file 5 — Supplementary Figure 4. [file 41598_2020_79385_MOESM5_ESM.pdf]

CD3\_170Er

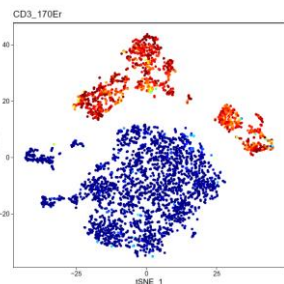

CD4\_174Yb

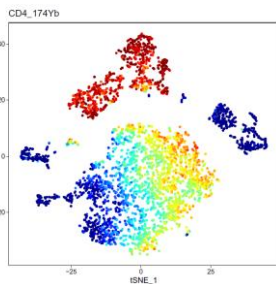

CD4\_146Nd

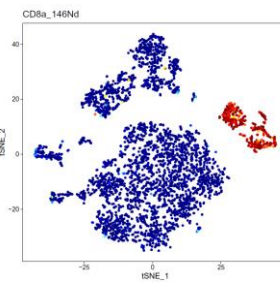

FOXP3\_159Tb

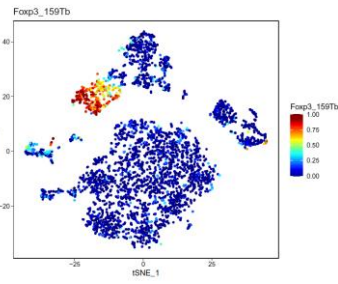

CD11c\_147Sm

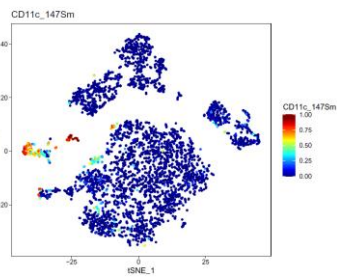

CD56\_166Er

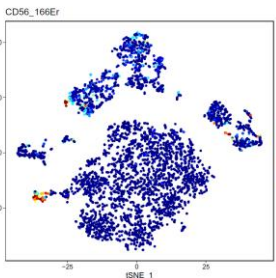

CD419\_142Nd

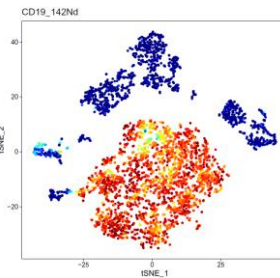

CD45\_89Y

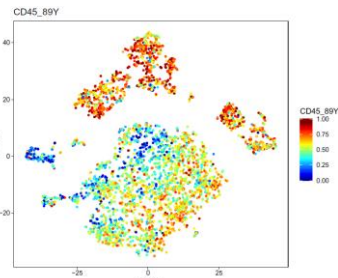

Figure S5

Supplement: Supplementary file 6 — Supplementary Figure 5. [file 41598_2020_79385_MOESM6_ESM.pdf]

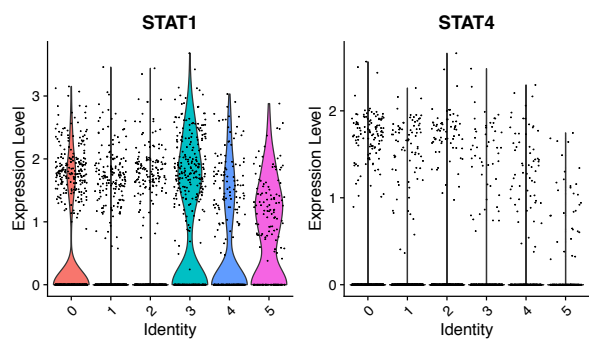

Sup Figure S6

Supplement: Supplementary file 7 — Supplementary Figure 6. [file 41598_2020_79385_MOESM7_ESM.pdf]
