# Supplementary material for: Potentiality of multiple modalities for single-cell analyses to evaluate the tumor microenvironment in clinical specimens
Source: Sci Rep. 2021 Jan 11;11:341. doi: 10.1038/s41598-020-79385-w (PMC7801605; doi:10.1038/s41598-020-79385-w)
Supplement: Supplementary file 8 — Supplementary Table 1. [file 41598_2020_79385_MOESM8_ESM.pdf]

Sup Table S1, Information of clinical samples used in Fig.1

Age	Sex	PS	Disease	Stage	Procedure	Related Figures
65	Male	0	Colorectal cancer	II	Surgery	Figure 1a, surgical resection, fresh and frozen
37	Male	0	Colorectal cancer	II	Surgery	Figure 1a, surgical resection, fresh and frozen
75	Male	0	Colorectal cancer	IIA	Suregry	Figure 1a, surgical resection, fresh and frozen
68	Female	0	Colorectal cancer	IIA	Suregry	Figure 1a, surgical resection, fresh and frozen
70	Male	0	Colorectal cancer	IIA	Suregry	Figure 1a, surgical resection, fresh and frozen
64	Female	0	Colorectal cancer	ⅢB	Biopsy	Figure 1a, biopsy, fresh
73	Male	0	Colorectal cancer	ⅢB	Biopsy	Figure 1a, biopsy, fresh
84	Male	0	Colorectal cancer	ⅡA	Biopsy	Figure 1a, biopsy, fresh
69	Male	0	Colorectal cancer	ⅣA	Biopsy	Figure 1a, biopsy, fresh
67	Female	0	Colorectal cancer	Ⅳ	Biopsy	Figure 1a, biopsy, fresh
75	Male	1	Gastric cancer	Ⅳ	Biopsy	Figure 1a, biopsy, frozen
46	Male	0	Gastric cancer	Ⅳ	Biopsy	Figure 1a, biopsy, frozen
83	Female	0	Gastric cancer	Ⅳ	Biopsy	Figure 1a, biopsy, frozen
73	Male	0	Gastric cancer	Ⅳ	Biopsy	Figure 1a, biopsy, frozen
56	Female	0	Gastric cancer	IV	Biopsy	Figure 1a, biopsy, frozen
