# Supplementary material for: Potentiality of multiple modalities for single-cell analyses to evaluate the tumor microenvironment in clinical specimens
Source: Sci Rep. 2021 Jan 11;11:341. doi: 10.1038/s41598-020-79385-w (PMC7801605; doi:10.1038/s41598-020-79385-w)
Supplement: Supplementary file 9 — Supplementary Table 2. [file 41598_2020_79385_MOESM9_ESM.pdf]

Sup Table S2, Information of clinical samples used in Fig.2, 3, 4, and 5

Case number	Age	Sex	PS	Disease	Stage	Procedure	Related Figures
gc_003	71	Male	0	Gastric cancer	IIIB	Surgery	Figure 2, 3, 4, and 5
gc_005	68	Female	0	Gastric cancer	IB	Surgery	Figure 2, 3, 4, and 5
gc_007	73	Male	0	Gastric cancer	IIIB	Suregry	Figure 2, 3, 4, and 5
gc_009	85	Male	0	Gastric cancer	IIIB	Suregry	Figure 2, 3, 4, and 5
gc_010	70	Female	0	Gastric cancer	IIIA	Suregry	Figure 2, 3, 4, and 5
