# Supplementary material for: Potentiality of multiple modalities for single-cell analyses to evaluate the tumor microenvironment in clinical specimens
Source: Sci Rep. 2021 Jan 11;11:341. doi: 10.1038/s41598-020-79385-w (PMC7801605; doi:10.1038/s41598-020-79385-w)
Supplement: Supplementary file 10 — Supplementary Table 3. [file 41598_2020_79385_MOESM10_ESM.pdf]

Sup Table S3, Antibodies used in the CyTOF

Metal	Molecule	Clone
89Y	CD45	HI30
141Pr	CD44	BJ18
142Nd	CD19	HIB19
143Nd	CD163	GHI/61
144Nd	CD45RA	HI100
145Nd		
146Nd	CD8a	RPA-T8
147Sm	CD11c	Bu15
148Nd	CD16	3G8
149Sm	CCR7	G043H7
150Nd	CD86	IT2.2
151Eu	Eomes	WD1928
152Sm	CD80	2D10.4
153Eu	PD-L1	29E.2A3
154Sm		
155Gd	PD-1	MIH4
156Gd	CD14	GCD14
158Gd	CD33	WM53
159Tb	FoxP3	236A/E7
160Gd		
161Dy	CTLA-4	14D3
162		
163Dy	Tbet	4B10
164Dy	Granzyme A	GB11
165Ho	CD28	CD28.2
166Er	CD56	NCAM16.2
167Er	Gata3	TWAJ
168Er		
169Tm	CD25	2A3
170Er	CD3	UCHT1
171Yb	Ki-67	Ki-67
172Yb		
173Yb	HLA-DR	SK3
174Yb	CD4	RPA-T4
175Lu		
176Yb	CD127	A019D5
209Bi	CD11b	ICRF44
