# Supplementary material for: Potentiality of multiple modalities for single-cell analyses to evaluate the tumor microenvironment in clinical specimens
Source: Sci Rep. 2021 Jan 11;11:341. doi: 10.1038/s41598-020-79385-w (PMC7801605; doi:10.1038/s41598-020-79385-w)
Supplement: Supplementary file 11 — Supplementary Table 4. [file 41598_2020_79385_MOESM11_ESM.pdf]

Sup Table S4. gene list used to annotate cell type

gene name	annotate to
CD3D	T cell
CD3E	T cell
CD4	CD4 T cell
CD8A	CD8 T cell
FOXP3	regulatory T cell
GNLY	NK cell
NCAM1	NK cell
NKG7	NK cell
CD79A	B cell
CD79B	B cell
MS4A1	B cell
CD19	B cell
CD27	Plasma
SDC1	Plasma
IL6R	Plasma
SLAM7	Plasma
IGHM	Plasma
IGHA1	Plasma
IGHA2	Plasma
IGHG1	Plasma
IGHG2	Plasma
IGHG3	Plasma
IGHG4	Plasma
CD14	Monocyte
FCGR3A	Monocyte
FCER1A	Dendric cell
CD1C	Dendric cell
EPCAM	Epithelial cell
VWF	Endothelial cell
CD34	Endothelial cell
MCAM	Endothelial cell
COL1A1	Fibroblast
COL1A2	Fibloblast
KIT	Mastcell
GATA2	Mastcell
HBA1	Erythrocyte
PTPRC	Immune cell
HSPA1B	Death
CD11c	Macrophage
CD68	Macrophage
