# Supplementary material for: Potentiality of multiple modalities for single-cell analyses to evaluate the tumor microenvironment in clinical specimens
Source: Sci Rep. 2021 Jan 11;11:341. doi: 10.1038/s41598-020-79385-w (PMC7801605; doi:10.1038/s41598-020-79385-w)
Supplement: Supplementary file 12 — Supplementary Table 5. [file 41598_2020_79385_MOESM12_ESM.pdf]

Sup Table S5, scRNA-seq (PBMC)

| | seq_id | Number of Reads | Reads Mapped Confidently to Transcriptome | Reads mapped confidently to intronic regions | Sequencing Saturation | Q30 bases in RNA read | number of cell after cut-off |
|---|---|---|---|---|---|---|---|
| gc_003 | s_029 | 127,837,255 | 51.90% | 31.60% | 79.40% | 91.00% | 2,671 |
| gc_005 | s_040 | 140,135,847 | 63.40% | 20.60% | 73.70% | 86.70% | 5,320 |
| gc_007 | s_045 | 138,436,653 | 63.10% | 23.60% | 92.20% | 90.50% | 1,515 |
| gc_009 | s_067 | 120,096,634 | 64.90% | 21.70% | 85.60% | 84.90% | 3,418 |
| gc_010 | s_075 | 43,860,040 | 58.30% | 27.00% | 85.80% | 89.90% | 941 |
