# Supplementary material for: Potentiality of multiple modalities for single-cell analyses to evaluate the tumor microenvironment in clinical specimens
Source: Sci Rep. 2021 Jan 11;11:341. doi: 10.1038/s41598-020-79385-w (PMC7801605; doi:10.1038/s41598-020-79385-w)
Supplement: Supplementary file 13 — Supplementary Table 6. [file 41598_2020_79385_MOESM13_ESM.pdf]

Sup Table S6. Differentially expressed genes in each Treg clusters in Figure 5b

Cluster	Gene	Avg. log FC	Adjusted P-value
0	TIGIT	0.71	1.50E-64
	MAGEH1	0.95	2.50E-61
	NGFRAP1	0.9	5.40E-51
	CARD16	0.62	6.40E-46
	CD27	0.55	1.70E-41
	CTSC	0.58	2.10E-36
	ACP5	0.63	5.80E-28
	RTKN2	0.57	2.00E-26
	PHLDA1	0.54	4.50E-23
	LAIR2	0.56	5.00E-23
1	CCL20	0.68	9.40E-41
	GPX1	0.59	3.70E-38
	GPR183	0.58	1.40E-30
	KLRB1	0.67	4.80E-30
2	CXCR4	0.86	7.00E-60
	KLF2	0.98	9.80E-59
	ZFP36L2	0.77	1.10E-37
	CCR7	0.68	3.00E-32
	CD69	0.58	3.00E-12
	PIK3R1	0.51	5.80E-07
3	IFI44L	0.79	1.10E-34
	CCL5	1.18	1.10E-26
	STAT1	0.62	1.10E-25
	ISG15	0.89	2.40E-24
	ALOX5AP	0.7	8.10E-23
	IFITM1	0.68	1.70E-22
	GIMAP4	0.6	2.50E-21
	NT5C3A	0.61	9.30E-21
	LY6E	0.51	4.50E-19
	MX1	0.67	6.00E-19
4	TNFRSF4	1.58	2.80E-92
	PTP4A3	1.13	8.10E-75
	TNFRSF18	1.08	7.90E-69
	PKM	0.91	7.60E-63
	LINC01588	0.71	3.10E-53
	TNFRSF9	1.06	3.20E-51
	EBI3	0.57	5.10E-43
	MIR155HG	0.74	1.80E-39
	SMS	0.79	2.70E-38
	GAPDH	0.58	3.20E-38
5	MKI67	1.61	7.00E-291
	GTSE1	0.85	4.30E-234
	KIAA0101	1.78	2.50E-233
	TOP2A	1.43	3.40E-233
	CENPF	1.32	4.20E-229
	BIRC5	1.06	4.40E-227
	RRM2	1.07	9.10E-216
	UBE2C	1.53	6.70E-214
	TYMS	1.21	1.30E-209
	CENPW	0.92	1.70E-206
