# Supplementary material for: Potentiality of multiple modalities for single-cell analyses to evaluate the tumor microenvironment in clinical specimens
Source: Sci Rep. 2021 Jan 11;11:341. doi: 10.1038/s41598-020-79385-w (PMC7801605; doi:10.1038/s41598-020-79385-w)
Supplement: Supplementary file 14 — Supplementary Table 7. [file 41598_2020_79385_MOESM14_ESM.pdf]

Sup Table S7, Stats of TIL scRNA-seq

	seq_id	Number of Reads	Reads Mapped Confidently to Transcriptome	Reads mapped confidently to intronic regions	Sequencing Saturation	Q30 bases in RNA read	number of cell after cut-off
gc_003	s_026	131,001,192	61.60%	21.70%	64.30%	91.10%	2,898
gc_005	s_034	129,466,618	68.70%	16.80%	61.20%	92.30%	11,714
	s_035	154,833,380	68.00%	17.40%	67.80%	90.80%	
	s_036	148,789,195	68.40%	16.70%	63.70%	89.80%	
	s_037	147,603,781	68.20%	16.70%	59.90%	89.30%	
gc007	s_041	168,686,343	68.20%	14.40%	44.00%	85.50%	7,462
	s_042	179,117,453	67.40%	14.70%	49.20%	86.30%	
	s_043	176,846,453	69.60%	13.50%	45.80%	84.70%	
	s_044	147,769,576	69.50%	14.30%	40.00%	91.20%	
gc_009	s_063	141,031,550	63.10%	19.90%	72.40%	79.60%	10,858
	s_064	127,884,737	62.70%	20.40%	75.60%	83.20%	
	s_065	157,390,174	65.00%	19.80%	67.70%	86.70%	
	s_066	144,745,148	63.50%	20.40%	64.30%	89.30%	
gc_010	s_071	141,020,433	54.90%	30.20%	79.50%	83.70%	11,711
	s_072	149,289,108	56.90%	28.60%	81.20%	84.20%	
	s_073	180,116,854	53.90%	27.30%	83.50%	77.60%	
	s_074	54,683,950	56.80%	27.50%	60.60%	89.40%	
